# Probabilistic maturation reaction norms assessed from mark–recaptures of wild fish in their natural habitat

**DOI:** 10.1002/ece3.1044

**Published:** 2014-04-01

**Authors:** Esben M Olsen, Dimitar Serbezov, Leif A Vøllestad

**Affiliations:** 1 Institute of Marine Research FlødevigenN-4817, His, Norway; 2 Centre for Ecological and Evolutionary Synthesis (CEES), Department of Biosciences, University of OsloP.O. Box 1066, Blindern, N-0316, Oslo, Norway; 3 Department of Natural Sciences, University of AgderP.O. Box 422, N-4604, Kristiansand, Norway

**Keywords:** Age and size at maturation, growth, life-history evolution, phenotypic plasticity, *Salmo trutta*, Survival

## Abstract

Reaction norms are a valuable tool in evolutionary biology. Lately, the probabilistic maturation reaction norm approach, describing probabilities of maturing at combinations of age and body size, has been much applied for testing whether phenotypic changes in exploited populations of fish are mainly plastic or involving an evolutionary component. However, due to typical field data limitations, with imperfect knowledge about individual life histories, this demographic method still needs to be assessed. Using 13 years of direct mark–recapture observations on individual growth and maturation in an intensively sampled population of brown trout (*Salmo trutta*), we show that the probabilistic maturation reaction norm approach may perform well even if the assumption of equal survival of juvenile and maturing fish does not hold. Earlier studies have pointed out that growth effects may confound the interpretation of shifts in maturation reaction norms, because this method in its basic form deals with body size rather than growth. In our case, however, we found that juvenile body size, rather than annual growth, was more strongly associated with maturation. Viewed against earlier studies, our results also underscore the challenges of generalizing life-history patterns among species and populations.

## Introduction

The age and body size at which organisms reach sexual maturity are key life-history traits potentially shaped by plastic responses to environmental conditions, as well as evolutionary responses to natural and anthropogenic selection (Reznick et al. 1990; Ernande et al. 2004; Gamelon et al. 2011). Hence, from both an evolutionary and a conservation perspective, there is a need to understand the underlying causes of phenotypic variation in maturation. In general, plastic responses are expected to shift the phenotype along a reaction norm, while an evolutionary response will shift the reaction norm itself (Hutchings 2011). Estimating such reaction norms is therefore a valuable tool in evolutionary biology.

Stearns and Koella (1986) used life-history theory to predict reaction norms for age and size at maturation and pointed out their potential to distinguish between genetic and plastic components of trait variation. Later, Heino et al. (2002) extended the method by specifically modeling the probabilistic nature of maturation. Since then, this probabilistic maturation reaction norm approach has been applied to time series on several exploited fish species, exploring to what extent temporal changes in maturation patterns may reflect fisheries-induced evolution versus phenotypic plasticity (Grift et al. 2003; Engelhard and Heino 2004; Olsen et al. 2004; Barot et al. 2005; Mollet et al. 2007).

Syntheses across studies revealed that probabilistic maturation reaction norms have declined (i.e., shifted toward maturation at smaller size for a given age) in many exploited fish populations and that the rate of change is correlated with the intensity of fishing, rates slowing down in cases where moratoria on fishing have been introduced by management authorities (Sharpe and Hendry 2009; Devine et al. 2012). This finding supports the long-held hypothesis that fishing may drive evolutionary changes in fish life histories (Miller 1957). However, the ability of probabilistic maturation reaction norms to detect evolution is still a matter of debate and investigation (Morita et al. 2009; Uusi-Heikkilä et al. 2011; Diaz Pauli and Heino 2013; Harney et al. 2013). By specifically modeling the probability of maturing at combinations of age and body size, probabilistic maturation reaction norms should be insensitive to any environmental variation (e.g., temperature or food availability) affecting maturation through growth plasticity (Heino et al. 2002). This is an important assumption for inferring about potential evolutionary changes in maturation. However, growth may not always be fully accounted for because a variety of juvenile growth trajectories may lead to a similar body size at age (Heino and Dieckmann 2008). For instance, growth during the most recent growing season, rather than body size, was the most important factor affecting the maturation of chum salmon (*Oncorhynchus keta*) during the subsequent season (Morita and Fukuwaka 2006). This supports the notion that – proximately – maturation will depend on the physiological state and rate of energy acquisition during critical periods (Thorpe et al. 1998), more closely related to growth than accumulated body size. Therefore, there is a need for detailed individual-based studies on the effects of growth histories on age and size at maturation in order to better disentangle phenotypic plasticity from evolutionary changes in exploited populations (Kuparinen et al. 2008). Furthermore, maturation reaction norms are typically retrospective by expressing the probability of maturing during a time interval (typically 1 year) against the body size and age reached at the end of that interval (Heino et al. 2002; Grift et al. 2003). Because the onset of maturation typically precedes the spawning season in teleost fishes (Kjesbu 1994; Tyler and Sumpter 1996; Thorpe 2007), a prospective approach using body size at the beginning of the interval may be preferred if such data are available.

Interpreting shifts in maturation reaction norms have a direct relevance for fisheries management and conservation. Young and small fish are often less productive compared to older and larger conspecifics (Trippel 1998; Berkeley et al. 2004), and a genetic shift in life histories toward maturation at a young age and small size could be very slow to reverse (Law 2000; but see Conover et al. 2009). Thus, genetic shifts in maturation reaction norms may lead to long-term economic as well as biological losses (Jørgensen et al. 2007; but see Eikeset et al. 2013).

While probabilistic maturation reaction norms have their limitations, it is important to know that they are tuned to the time series datasets that usually exist for harvested fish stocks, that is, destructive sampling with basic information about individual fish age, size, and maturity state. Direct observations on age and size at maturation are often not available for wild fish in their natural habitat. For instance, when individual life histories cannot be tracked over time, first-time spawners may be confused with fish that have spawned during multiple years. Typically, probabilistic maturation reaction norms are therefore based on an indirect approach comparing proportions of mature individuals at age and size during consecutive years (Barot et al. 2004). This demographic method rests on two assumptions: that immature and mature individuals have equal age-specific growth- and survival rates (Barot et al. 2004). Expected life-history trade-offs associated with reproduction in natural populations suggest that these assumptions will often not hold (Stearns 1992). However, few studies have actually investigated the survival cost of reproduction in fishes (Kuparinen et al. 2012; but see Hutchings 1994). A negative correlation between reproductive activity and somatic growth rate has been observed in coral reef fish (Warner 1984). When sample sizes are large (>500), simulation studies suggest that the demographic reaction norm method is robust to violations of these assumptions (Barot et al. 2004). However, significant biases may occur when sample sizes are smaller. For instance, a relatively high mortality of maturing fish may result in overestimation of the maturation reaction norm midpoints, that is, the combinations of age and body length where an individual reaches a 50% probability of maturing (Barot et al. 2004).

Mark–recapture techniques potentially allow for monitoring individual life histories through time in natural habitats. By providing direct observations on body size, growth, and maturation, mark–recapture data can be used to assess strengths and limitations of the demographic maturation reaction norm approach. Here, we estimated probabilistic maturation reaction norms based on 13 years of mark–recapture data on a salmonid fish, the brown trout (*Salmo trutta*). To our knowledge, this is the first time that maturation reaction norms have been quantified based on such direct observations of individual fish life-history trajectories in the wild. Essentially, the direct observations of fish captured as juveniles at one age and then recaptured, either as a juveniles or spawners, the year after allowed us to model the probability of maturing instead of simply the probability of being mature. This distinction is important because the latter describes a state which does not distinguish between newly matured fish and fish that have spawned at an earlier age, while the former describes the actual life-history transition – the process of maturation – during a given time interval (Heino and Dieckmann 2008). The mark–recapture data also allowed us to directly evaluate the influence of juvenile growth history and body size on the probability of maturing. First, we ask whether the probability of maturing at size and age is best explained by body size at the end of the interval where maturation may occur (retrospective approach) or, alternatively, by body size at the beginning of the time interval (prospective approach). Second, we ask to what extent body size or, alternatively, juvenile growth history (length increment during the year before maturation) explains patterns in maturation. Lastly, we assess the demographic maturation reaction norm approach (Barot et al. 2004) by comparing with maturation reaction norm estimates based on direct observations from mark–recaptures. As part of this assessment, we also evaluate the assumptions of similar growth- and survival rates for juvenile and maturing fish within an age class. We discuss how these results may improve our understanding of the strengths and limitations of the probabilistic maturation reaction norm approach as a tool in evolutionary biology.

## Materials and Methods

**Figure 1:**
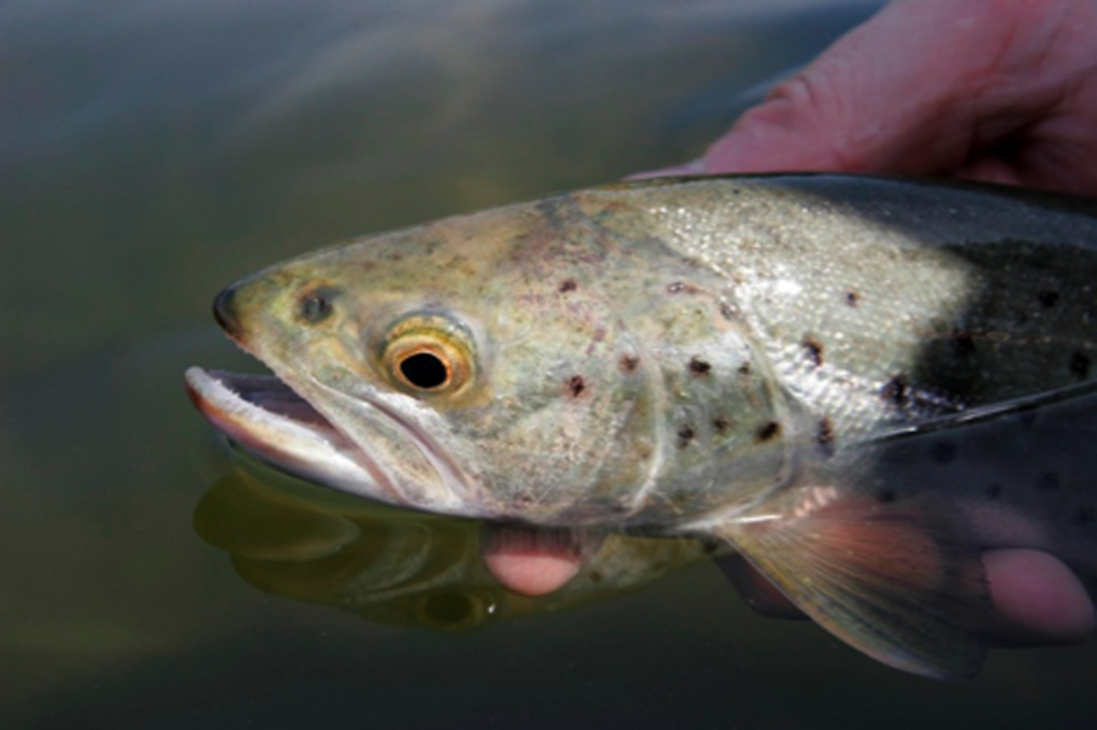
Brown trout (*Salmo trutta*). Photograph by Øystein Paulsen used with permission.

Brown trout (Fig. 1) is an iteroparous fish that breeds in fresh water during autumn, usually in streams or rivers. In some populations, trout remain in their natal freshwater habitat throughout their lives, whereas other populations are characterized by feeding migrations into lakes or the ocean (Jonsson and Jonsson 2011). The species is native to Europe but has been introduced throughout large parts of the world (Budy et al. 2013). We sampled trout from a stream-resident population in eastern Norway (the Bellbekken stream; 61°15′N, 11°51′E) during 13 consecutive spawning seasons (1997–2009). Trout density in this stream is relatively low, and the fish rarely grow beyond 6 years, 20 cm and 100 g (Olsen and Vøllestad 2005; Bærum et al. 2013). Fishermen seldom visit the stream. Trout demography in this system is strongly influenced by stochastic factors, but density-dependent processes also play a role (Carlson et al. 2008). Both polygamous and monogamous matings occur, and there is a large reproductive skew for both sexes (Serbezov et al. 2010, 2012).

We used a backpack electrofishing apparatus to capture trout within twenty-five contiguous and permanent stream sections spanning 1.5 km in total (for details, see Bærum et al. 2013). The total population size (excluding age 0 fish) in this study area is about 900–1400 trout (Serbezov et al. 2010). All captured trout were measured (mm fork length) and classified as either juvenile or mature based on external characteristics. Mature females were recognized from their rounded soft abdomen and protruding genital opening. A fish was classified as a mature male if milt was released when gently pressing the abdomen. Also, these male fish had a thick, slimy skin with scales deeply embedded in it, a likely adaptation to prevent fighting injuries at the spawning sites (Fleming 1996). The authors have experience in recognizing mature females and males from an earlier study when fish from this stream were euthanized and dissected to confirm maturity state (Olsen and Vøllestad 2005). At first capture, all fish (excluding most age 0 juveniles) were individually tagged using passive integrated transponder tags (Prentice et al. 1990) or visible implant elastomer marks (Olsen and Vøllestad 2001), and a few scales were removed for age determination (Morita and Fukuwaka 2006). A fish was considered to be age 0 from hatching in spring (April–May) until the next 1 January, age 1 during its second year of life, and so on. After handling, the fish were allowed to recover and then released at the site of capture. Recapture probabilities were usually around 0.4–0.7 (Carlson et al. 2008), indicating that we were able to capture a fairly large proportion of the fish population in the stream each year.

**Figure 2:**
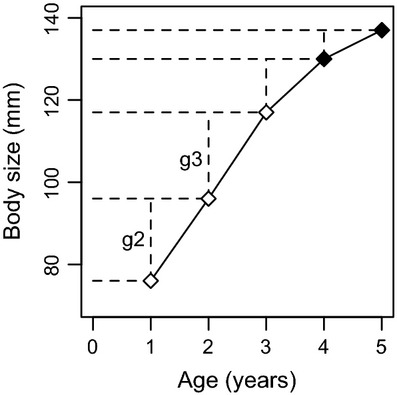
Capture and recapture of a female trout in the Bellbekken stream, eastern Norway, during the 2004−2008 spawning seasons, illustrating the method of direct observations of juvenile growth (mm year^−1^; g2: age 1–2 interval, g3: age 2–3) and maturation (open = juvenile, filled = mature) used to assess the probabilistic maturation reaction norm approach.

Based on this sampling regime, we constructed capture histories for each fish, with information on length and maturity state (juvenile or spawner) at each capture and recapture occasion (Fig. 2). We have previously used this dataset to estimate growth and survival (Carlson et al. 2008; Bærum et al. 2013). Here, we used a subset of the data for an evaluation of the probabilistic maturation reaction norm approach through direct observations of individual growth and maturation. First, we included all fish captured at age 2 (*N* = 1238, mean length: 100 mm, range: 66–155 mm) because this is the youngest age where maturation takes place in this population (Olsen and Vøllestad 2005). Hence, any fish seen as mature at age 2 would be a first-time spawner. A subset of these age 2 fish were also observed at age 1 (*N* = 343, mean length: 77 mm, range: 56–122 mm) allowing observations on growth from age 1 to age 2. Thus, annual growth was quantified as the observed increase in fork length for trout captured and recaptured during consecutive years. Second, we included all fish seen as juvenile at age 2 and then observed again at age 3, either as a juvenile or spawner (*N* = 348, mean length: 121 mm, range: 88–159 mm). This allowed us to directly estimate maturation at age 3. Similarly, we analyzed maturation at age 4 from fish seen as juvenile at both age 2 and age 3 and then seen again as juvenile or mature fish at age 4 (*N* = 117, mean length: 139 mm, range: 107–168 mm). Age 5 and older fish were sparse and not included in the analyses.

The data were analyzed using generalized linear models (McCullagh and Nelder 1989) with maturation state as binary response variable and individual growth and body size as predictor variables. Akaike's information criterion (AIC) was used to compare the performance of alternative candidate models (Burnham and Anderson 1998). The model with the smallest AIC value will represent the best compromise between lack of precision (including too many parameters) and bias (too few parameters). Also, AIC can be used to compare alternative models that are not nested. First, we compared a retrospective approach to a prospective approach by modeling the probability of maturing as a function of body size during the current or previous year of sampling:


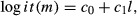
1

where *m* is the probability of maturing at age 3 years, *c*_0_ is the intercept, and *l* is the body size (mm fork length) at age 3 (retrospective approach) or age 2 (prospective approach). Similarly, the probability of maturing at age 4 was modeled as a function of body size at age 4 or age 3.

Second, we explored whether growth history, rather than body size, is better at predicting the probability of maturing at age:


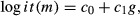
2

where *m* is the probability of maturing at age 3 years, *c*_0_ is the intercept, and *g* is growth (mm) from age 1 to age 2. Similarly, the probability of maturing at age 4 was modeled as a function of growth from age 2 to age 3. These growth history models aim to quantify an effect of growth during year_*i*_ on the probability of spawning in year_*i* + 1_. The rationale for this is that we expect the decision to mature will take place well in advance of the actual spawning season (Tyler and Sumpter 1996). We did not test for an effect of growth during the same season where maturation is estimated. At this point, growth rate may be influenced by the allocation of energy to gonads. The trade-off between maturation and growth is explored in a separate model (see below). For comparison among models and other studies, the growth and size variables were standardized to a mean of zero and a standard deviation of unity within each age class. However, the demographic method and direct method of estimating probabilistic maturation reaction norms were compared using unstandardized data, allowing the most direct visual interpretation of the reaction norms.

Third, we compared probabilistic maturation reaction norms estimated directly from our mark–recapture approach to the demographic approach often applied to field data in cases where direct information about the maturation event is not available (i.e., where repeat spawners cannot be distinguished from first-time spawners). Following Barot et al. (2004), the probability of maturing at age can then be estimated from:


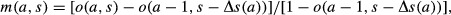
3

where *m*(*a*,*s*) is the probability of maturing at age *a* and size *s*, *o*(*a*,*s*) is the probability of being mature (including both first-time spawners and repeat spawners) at age *a* and size *s*, and Δ*s* the average length increment from age *a* − 1 to age *a*. Here, *m* is defined as the retrospective probability of having matured during the year leading up to age *a* when the fish is sampled. First, we estimated annual growth as the difference in mean body length between two consecutive ages. Next, the probabilities of being mature at combinations of age and length (needed for equation 3) were estimated from:


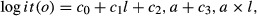
4

where age is modeled as a factor. The probabilities of maturing at age and length were then calculated from equation (3) using the predictions for being mature estimated from equation (4). The probability of being mature at age 1 was set to zero, because no mature fish were observed at this age. Lastly, reaction norm midpoints (the combinations of age and size where the probability of maturing reaches 0.5, *L*_*P*50_) were estimated for each age group from a model similar to equation (1) and substituting 0.5 for *m* (for details, see Barot et al. 2004). To avoid pseudoreplication, each fish was only used once, the last time it was observed. We then compared these reaction norm midpoint estimates to estimates derived from our retrospective mark–recapture approach (equation 1). For both approaches, we simplified the statistical modeling by pooling different cohorts, females and males. Our data did not permit the estimation of separate maturation reaction norms for females and males, because we could not determine the sex of those individuals that were only observed as juveniles. All fish were released alive, and spawners were identified and sexed based on external characteristics. However, in our study system, females and males do seem to have similar maturation schedules (Olsen and Vøllestad 2005). Also, the method of Barot et al. (2004) requires relatively large sample sizes, hence the need to simplify models (Heino and Dieckmann 2008).

As part of our assessment, we used the mark–recapture information to evaluate the assumptions of similar age-specific growth and survival rates. First, we used a linear model to test for an effect of maturation at age 3 on growth rate from age 2 to age 3 (the time interval where resources are allocated to maturing gonads):


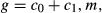
5where *g* is growth rate (annual length increment) and *m* is maturity state (juvenile or spawner). A similar model was used to test for an effect of maturation at age 4 on growth rate from age 3 to age 4. Second, we used a generalized linear model to test for an effect of maturation at age 3 on survival from age 3 and onwards:


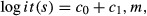
6

where *s* is the probability of surviving beyond age 3, and *m* is the maturity state at age 3. Following Carlson et al. (2008), survival was modeled as a binary variable where a fish was classified as survived if seen again (recaptured) after age 3 and dead if never seen again. A similar model was used to test for an effect of maturation at age 4 on survival from age 4 and onwards.

## Results

**Figure 3:**
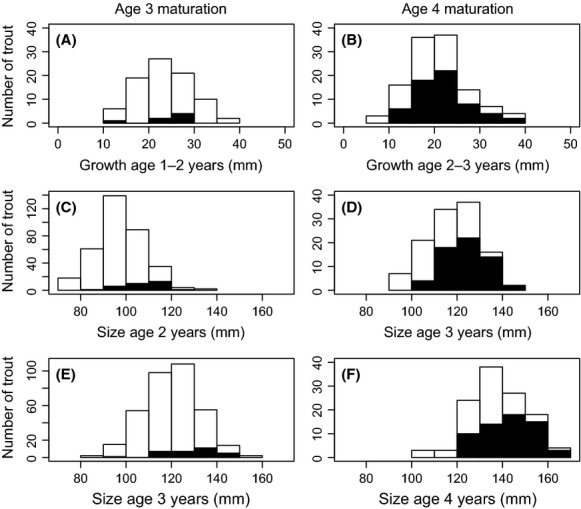
Trout growth rates (mm year^−1^), body size (mm), and maturation as observed from individual mark–recaptures in the Bellbekken stream, eastern Norway, during 1997–2009. Left histograms show the number of juvenile (open) and maturing (filled) trout at age 3 in relation to growth during age 1–2 (A), body size at age 2 (C) and body size at age 3 (E). Right histograms show maturation at age 4 in relation to age 2–3 growth (B), size at age 3 (D), and size at age 4 (F).

Trout that were larger at age 3 had a higher probability of maturing at this age compared to smaller age 3 trout (model 1: *c*_1_ = 0.86, SE = 0.21, *P* < 0.001, Fig. 3). Similarly, trout that were larger at age 2 also had a higher probability of maturing at age 3 (model 1: *c*_1_ = 1.10, SE = 0.22, *P* < 0.001, Fig. 3). In terms of AIC, the prospective model having body size at age 2 as predictor of maturation at age 3 outperformed the retrospective model based on age 3 body size (ΔAIC = 12.45). For the subset of age 3 fish with information about growth during the age 1 to age 2 interval (*N* = 85), there was no significant effect of growth history on the probability of maturing at age 3 (model 2: *c*_1_ = 0.13, SE = 0.40, *P* = 0.75, Fig. 3). The positive effect of body size at age 2 on the probability of maturing at age 3 was, however, maintained for this subset of data (model 1: *c*_1_ = 1.18, SE = 0.49, *P* = 0.016).

Results were similar for maturation at age 4. Larger age 4 fish had a higher probability of maturing at this age compared to smaller fish (model 1: *c*_1_ = 0.90, SE = 0.24, *P* < 0.001, Fig. 3). Trout that were larger at age 3 also had a higher probability of maturing at age 4 (model 1: *c*_1_ = 1.23, SE = 0.27, *P* < 0.001, Fig. 3). There was a marginally significant effect of fish growth during the age 2 to age 3 interval on the probability of maturing at age 4 (model 2: *c*_1_ = 0.39, SE = 0.20, *P* = 0.047, Fig. 3). In terms of AIC, the prospective model containing age 3 body size as explanatory variable outperformed both the retrospective size model (ΔAIC = 11.90) and the growth history model (ΔAIC = 26.00).

**Figure 4:**
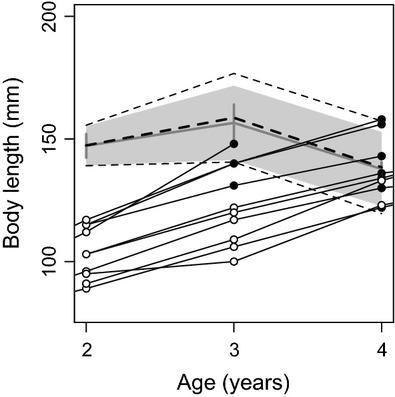
Trout probabilistic maturation reaction norm, showing the reaction norm midpoint (*L*_*P*__50_, gray line, SE: horizontal lines) and envelope (*L*_*P*__25_–*L*_*P*__75_, gray polygon) estimated from individual growth and maturation trajectories. Individual life histories were observed directly from mark–recaptures, illustrated here with ten individuals (open circle = juvenile, filled circle = mature). For comparison, reaction norm midpoints and envelope (dashed lines) were also estimated using the demographic approach developed by Barot et al. (2004).

The probabilistic maturation reaction norm estimated directly from the mark–recapture data was relatively flat (weakly positive) between age 2 and age 3, while the slope was negative between age 3 and age 4 (Fig. 4). This last result implies that the body size at which the trout reach a given probability of maturing will decrease with age. The probabilistic maturation reaction norm based on the demographic method (Barot et al. 2004) displayed a similar shape and position (Fig. 4). In particular, the midpoint (*L*_*P*50_) estimates were very similar between the two approaches, while the mark–recapture direct approach resulted in a slightly narrower maturation envelope (i.e., the *L*_*P*25_–*L*_*P*75_ interval, Fig. 4). For example, at age 3, the mark–recapture maturation envelope spanned 30 mm, while the demographic maturation envelope spanned 36 mm (a 20% increase, Fig. 4).

There was no significant effect of maturation on growth rate during the year leading up to the spawning season, although slope estimates were negative (model 5: age 3: *c*_1_ = −0.21, SE = 0.19, *P* = 0.28; age 4: *c*_1_ = −0.21, SE = 0.19, *P* = 0.27). There was a negative effect of maturation at age 3 on the probability of being seen again (surviving) at older ages (model 6: *c*_1_ = −1.17, SE = 0.47, *P* = 0.013). Only 19% of fish maturing at age 3 were seen again at older ages, while 44% of age 3 juveniles were seen again. In contrast, there was no significant effect of maturation at age 4 on the probability of being seen again at older ages (model 6: *c*_1_ = −0.16, SE = 0.41, *P* = 0.71). A total of 27% of fish maturing at age 4 were seen again at older ages, while 30% of age 4 juveniles were seen again.

## Discussion

By direct observations of growth and maturation of wild fish in their natural habitat, this study evaluates the probabilistic maturation reaction norm approach as a tool in evolutionary biology. Most importantly, we found only minor differences in the shape and position of reaction norms when comparing our direct approach to the much applied demographic approach developed by Barot et al. (2004). Our study also showed that body size measured at the previous age (a prospective approach) is a more important determinant of maturation than body size measured at current age (a retrospective approach). Interestingly, we found that body size was also a more important determinant of maturation than previous growth history. We discuss these results against the current understanding and debate on the role of probabilistic maturation reaction norms in distinguishing evolutionary changes from phenotypic plasticity, particularly in cases where harvesting by humans (e.g., fisheries) is causing rapid phenotypic changes in the exploited populations (Darimont et al. 2009).

Encouragingly, our study suggests that probabilistic maturation reaction norms estimated without direct knowledge about individual maturation and growth patterns (Barot et al. 2004) can nevertheless be quite precise. In our case, adding the direct mark–recapture information about individual life histories had only minor influence on the slope or position of the probabilistic maturation reaction norm. For comparison, Pérez-Rodríguez et al. (2009) were able to pinpoint Atlantic cod (*Gadus morhua*) age and size at maturation from histological analyses of gonads (separating recruit spawners and repeat spawners) and concluded that this information did not significantly change the probabilistic maturation reaction norms, which were highly correlated with estimates based on the demographic (Barot et al. 2004) approach. We are not aware of other similar studies that have assessed the demographic maturation reaction norm approach by adding more direct information about the maturation process. However, several studies have pointed out strengths and limitations of the demographic maturation reaction norm approach and suggested modifications and alternatives (Dieckmann and Heino 2007; Marshall and McAdam 2007; Heino and Dieckmann 2008). For example, Van Dooren et al. (2005) developed a rate-based maturation model applicable to data with no typical periodicity (e.g., annual spawning), while Uusi-Heikkilä et al. (2011) demonstrated the significance of fish condition as an additional dimension of probabilistic maturation reaction norms.

For fisheries research, our study and the study by Pérez-Rodríguez et al. (2009) are good news, because they suggest that basic information about fish age, size, and maturity state can provide important information about life-history processes that are relevant for fisheries management and conservation. For instance, temporal changes in maturation reaction norms may serve as early warning signals of populations at risk (Olsen et al. 2005; see also, Trippel 1995).

Our mark–recapture data also revealed that the assumption of equal survival rates between juvenile and mature fish of the same age did not hold, because fish maturing at age 3 had lower postspawning survival than juveniles of the same age. This indicates a survival cost of reproduction in the trout population. However, there was no clear evidence for a cost of reproduction in terms of reduced growth rates, because growth did not differ significantly between juvenile and maturing fish in the time period when energy is allocated to gonads. These findings are in accordance with earlier simulation results, suggesting that errors in maturation reaction norm estimates should be more sensitive to a violation of the assumption of equal growth rates compared to survival rates (Barot et al. 2004). We note that the percentage of fish seen again after initial release will underestimate true survival if some fish remain alive within the study area but are never recaptured, and also if some fish disperse permanently from the study area but remain alive in another area (Lebreton et al. 1992; Ergon and Gardner 2014). Earlier, we documented a high year-specific probability of recapturing tagged trout in our study stream (Carlson et al. 2008). This probably relates to the fact that the stream is small and easy to sample and that Bellbekken trout are stationary to the extent that there is evidence for isolation by distance genetic structure within our study area and very limited movement beyond the study area (Carlson et al. 2008; Vøllestad et al. 2012). A potential bias in survival might remain if maturing fish have a different recapture probability compared to juvenile fish.

In general, life-history trade-offs between reproduction, growth and survival are expected from theory (Stearns 1992; Roff et al. 2006) but may be difficult to detect in natural systems (Hamel et al. 2014). Individuals may differ in how much resources they have available, which can mask the expected negative correlations between life-history traits (van Noordwijk and de Jong 1986). In our study, the survival cost of reproduction was only seen at age 3 and not at age 4. Also, fish that delayed maturation beyond age 4 had relatively low survival compared to age 3 juveniles. It is possible, therefore, that these late-maturing fish were simply of poor quality or had poor access to resources, such as overwintering habitats. Hutchings (1994) found evidence for a survival cost of reproduction in brook trout (*Salvelinus fontinalis*), showing that this cost was higher for older fish (attributed to a senescent decline in body condition). While this result seems to contradict our finding, we only estimated survival at a young and intermediate age, while data on older fish were too sparse to be included in the analyses. Interestingly, Descamps et al. (2009) found no evidence for a survival cost of reproduction in prime-aged red squirrels (*Tamiasciurus hudsonicus*) but did detect a negative effect of breeding on survival for the youngest and the older squirrels, possibly related to a sharper trade-off with growth for the young individuals and senescence in the old individuals.

In our trout population, body size in the beginning of the maturation time interval (previous year, a prospective approach) was a better predictor of maturation compared to body size measured at the end of the time interval (a retrospective approach). This is perhaps not surprising, because the allocation of energy to gonads starts months ahead of the actual spawning (Tyler and Sumpter 1996). While demographic maturation reaction norms are typically estimated from a retrospective approach (Grift et al. 2003), our study speaks in favor of adding information about body size earlier in life (see also, Diaz Pauli and Heino 2013; Harney et al. 2013). We acknowledge that information about the juvenile life history will often not be directly available from collected data. Conducting mark–recapture studies on mobile species in open aquatic environments can no doubt be a challenging task. Still, recent studies show that marine fish such as the Atlantic cod can be structured into genetically distinct local populations at a surprisingly small geographic scale, with limited movement of juvenile and mature fish allowing for mark–recapture estimates of life-history traits, selection processes, and population sizes (Knutsen et al. 2011; Olsen and Moland 2011; see also, DiBattista et al. 2011). Also, an indirect approach using back-calculated body size from fish scales or otoliths may serve as a good alternative (Morita and Fukuwaka 2006).

We found that previous body size was a better predictor of maturation than previous growth rate. This result has important implications for the interpretation of shifts in maturation reaction norms in time or space. Traditional maturation reaction norms do not directly account for effects of previous growth history, only the end result, which is body size at age (Heino et al. 2002). The important study by Morita and Fukuwaka (2006) showed that previous growth history was more closely linked to maturation in chum salmon than previous body size and that growth-driven plasticity could therefore significantly influence maturation reaction norm estimates (see also, Kuparinen et al. 2008). In our study, on the other hand, body size was the more important variable. We do not know why this is so, but note that the chum salmon is an anadromous species where rapid growth takes place after migrating from freshwater to the ocean. In contrast, our brown trout population is stream-resident and do not show the same age-specific increase in growth rate (Bærum et al. 2013).

In conclusion, our empirical assessment shows that the probabilistic maturation reaction norm approach may perform well even if the key assumption of equal survival between juvenile and maturing fish does not hold. The observation that juvenile body size, rather than growth, was more strongly associated with maturation, underscores the need to understand how life-history traits are linked for each specific species or population subject to reaction norm analyses.
